# Plasma p-Cresol Lowering Effect of Sevelamer in Peritoneal Dialysis Patients: Evidence from a Cross-Sectional Observational Study

**DOI:** 10.1371/journal.pone.0073558

**Published:** 2013-08-28

**Authors:** Bruna Guida, Mauro Cataldi, Eleonora Riccio, Lucia Grumetto, Andrea Pota, Silvio Borrelli, Andrea Memoli, Francesco Barbato, Gennaro Argentino, Giuliana Salerno, Bruno Memoli

**Affiliations:** 1 Division of Physiology, Dept. of Clinical Medicine and Surgery, Federico II University of Naples, Naples, Italy; 2 Division of Pharmacology, Dept. of Neuroscience, Reproductive and Odontostomatologic Sciences, Federico II University of Naples, Naples, Italy; 3 Dept. of Nephrology, Federico II University of Naples, Naples, Italy; 4 Dept. of Pharmaceutical and Toxicological Chemistry, Federico II University of Naples, Naples, Italy; 5 Dept. of Biochemistry and Medical Biotechnology, Federico II University of Naples, Naples, Italy; 6 Nephrology Division, Second University of Naples, Naples, Italy; Universidade de São Paulo, Brazil

## Abstract

p-Cresol is a by-product of the metabolism of aromatic aminoacid operated by resident intestinal bacteria. In patients with chronic kidney disease, the accumulation of p-cresol and of its metabolite p-cresyl-sulphate causes endothelial dysfunction and ultimately increases the cardiovascular risk of these patients. Therapeutic strategies to reduce plasma p-cresol levels are highly demanded but not available yet. Because it has been reported that the phosphate binder sevelamer sequesters p-cresol *in vitro* we hypothesized that it could do so also in peritoneal dialysis patients. To explore this hypothesis we measured total cresol plasma concentrations in 57 patients with end-stage renal disease on peritoneal dialysis, 29 receiving sevelamer for the treatment of hyperphosphatemia and 28 patients not assuming this drug. Among the patients not assuming sevelamer, 16 were treated with lanthanum whereas the remaining 12 received no drug because they were not hyperphosphatemic. Patients receiving sevelamer had plasma p-cresol and serum high sensitivity C-reactive protein concentrations significantly lower than those receiving lanthanum or no drug. Conversely, no difference was observed among the different groups either in residual glomerular filtration rate, total weekly dialysis dose, total clearance, urine volume, protein catabolic rate, serum albumin or serum phosphate levels. Multiple linear regression analysis showed that none of these variables predicted plasma p-cresol concentrations that, instead, negatively correlated with the use of sevelamer. These results suggest that sevelamer could be an effective strategy to lower p-cresol circulating levels in peritoneal dialysis patients in which it could also favorably affect cardiovascular risk because of its anti-inflammatory effect.

## Introduction

Uremic toxins are a heterogeneous group of compounds that accumulate in the plasma of patients with chronic kidney disease (CKD). More than 90 different uremic toxins have been identified up to day; based on their molecular weight and their affinity for plasma proteins, they can be classified in three different groups: small water soluble molecules not significantly bound to plasma proteins, small molecules significantly bound to plasma proteins and middle/large proteins [1]. The great interest that has been accruing on uremic toxins over the years derives from experimental evidence suggesting that some of them may have a causative role in the development of long-term complications of CKD and, in particular, of cardiovascular disorders [2], which are major cause of death in this disease [3]. Recent evidence points to p-cresol as one of the uremic toxins more directly implicated in the pathogenesis of CKD complications. This aromatic compound is generated in the gut by the degradation of tyrosine and phenylalanine operated by resident intestinal flora [4]–[5]. After absorption, p-cresol is converted into its conjugates p-cresylglucuronide and p-cresylsulfate. The latter, which represents more than 95% of circulating p-cresol, is responsible for the majority of p-cresol toxic effects [6]. The plasma concentrations of p-cresol and p-cresylsufate, which belong to the subgroup of small molecules significantly bound to plasma proteins, are strongly related to cardiovascular risk in CKD [6]–[9] and are predictive of mortality in these patients [10]. This is consistent with a number of studies *in vitro* that clearly showed that p-cresol and its derivative p-cresylsulphate are toxic for endothelial cells and can cause endothelial dysfunction [11]–[13]. Intense efforts are currently directed to identify the best therapeutic strategy to lower uremic toxins in CKD patients because it has been shown that this can lead to a significant improvement in their survival [14]. Unfortunately, dialysis seems to be effective only in removing small water soluble uremic toxins whereas those significantly bound to plasma proteins are significantly retained despite the dialysis treatment [15]. Specifically, p-cresol and its sulphate derivative are extremely difficult to dialyze [16]. An interesting alternative approach to lower the plasma concentrations of p-cresol is directed to lowering its intestinal absorption [17]. The rationale behind this strategy is that all circulating p-cresol is derived from that produced by bacteria in the gut because this compound cannot be generated by the metabolism of aromatic aminoacids by human cells [17]. Studies *in vitro* showed that the non-calcium non-aluminum containing phosphate binder sevelamer hydrochloride (*Sev*), which is largely used to treat hyperphosphatemia in end stage renal disease (ESRD) [18]–[19], also binds p-cresol [20]. This evidence suggested that this orally administered phosphate binder could lower p-cresol concentrations in human patients with CKD by preventing its intestinal absorption. Contrarily to these expectations, Brandeburg et al. (2010) [21] reported that p-cresol plasma concentrations were significantly higher at the end of an 8 week treatment with *Sev* than before it was started and, importantly, that they returned at their basal levels when the treatment with this drug was stopped. However, this remains the only study that explored the effects of *Sev* on p-cresol in hemodialysis patients. In addition, the impact of the treatment with this drug on p-cresol levels in peritoneal dialysis (PD) patients has never been investigated. Considering this lack of information, in the present cross-sectional observational study, performed on a cohort of 57 patients with ESRD treated with PD, we compared p-cresol plasma concentrations in patients assuming *Sev* for the treatment of ESRD-induced hyperphosphatemia and in those not treated with this drug.

## Patients and Methods

### Study Design

The present study has a monocentric cross-sectional observational design. All the patients undergoing PD at the Division of Nephrology of the Federico II University of Naples were considered for recruitment. The *inclusion criteria* were: age >18 years, dialysis age >6 months, a good compliance to medical and dialysis treatment, and, for the patients assuming *Sev* or lanthanum, a stable therapeutic regimen with either of these phosphate binders from at least six months. *Exclusion criteria* were: malnutrition, malignant neoplasms, and current history of gastrointestinal and/or endocrine diseases.

All subjects gave their written informed consent to participate the study that was performed in accordance with the indications of the WMA Declaration of Helsinki. As required by Italian regulations governing observational studies (AIFA document of 20/3/2008), a formal notification of the study was sent to the Ethics Committee of the Federico II University of Naples.

Detailed demographic and clinical history data were already available for each patient at the time of recruitment as their collection is part of the standard protocol applied for all the patients followed at our institution. Blood samples for the determination of blood chemistry and of p-cresol were collected in the morning from all patients recruited for the study. For data analysis, we divided patients into three groups: patients assuming *Sev* (n = 29), patients assuming lanthanum (n = 16) and patients not assuming any phosphate binder (no binder) (n = 12) . Importantly, at our institution, the choice of the drug (*Sev* or lanthanum) to be used in hyperphosphatemic patients is left entirely free to the medical doctor taking care of these patients. Therefore no explicit bias was introduced in the composition of the *Sev* and lanthanum subgroups by “a priori” selection criteria. The dose of either lanthanum or *Sev* was adjusted, as usual, to the target PO_4_ and, therefore, different doses were used in different patients.

### Chemistries

The following blood chemistries were determined by standard laboratory procedures in the venous blood samples collected from our patients: urea nitrogen, creatinine, bicarbonate, Na^+^, K^+^, PO_4_, Ca^2+^, intact PTH (iPTH), albumin, total cholesterol, and high density lipoprotein (HDL)-cholesterol, triglycerides, hemoglobin, glycosylated hemoglobin (HbA1c) and high sensibility C-reactive protein (hs-CRP). Urea nitrogen, creatinine, Na^+^, K^+^, PO_4_, and proteins were also measured in samples from 24 h urine specimens and from peritoneal effluent.

In all the recruited patients, residual glomerular filtration rate (rGFR), total, renal and peritoneal creatinine clearance, normalized protein catabolic rate (nPCR) and total, renal and peritoneal weekly dialysis dose (Kt/V) were evaluated at the time of the study.

### p-Cresol assay

p-Cresol plasma concentrations were determined by a slightly modified version of the HPLC method proposed by De Smet et al. [22]. This method involves a preliminary acidification of the plasma sample to release bound p-cresol conjugates from plasma proteins, so that total (bound + free) p-cresol-conjugates become available for further HPLC analysis. Plasma acidification also causes the hydrolysis of both p-cresylsulphate and p-cresylglucuronide that are converted in p-cresol. Therefore, what is measured by this method is mainly p-cresylsulfate [6]. Briefly, the experimental procedure was performed as follows: plasma samples (300 µl) were acidified by adding half a volume of 25% w/v perchloric acid (150 µl) to release p-cresol from plasma proteins. After 10 s vortexing, 600 µl of ethyl acetate was added to the sample to extract p-cresol and the resulting mixture was saturated with 100 mg of NaCl and vortexed for 10 s. The sample was then centrifuged at 865 *g* for 5 minutes and the supernatant containing p-cresol was collected in a new tube and centrifuged again at the same speed. The clear supernatant obtained after this second centrifugation was collected and injected on a reversed-phase Ascentis C18 HPLC column with a Supelguard Ascentis C18, guard column (both from Supelco, Bellefonte, PA, USA) mounted on a LC-10AD VP HPLC apparatus (Shimadzu- Corporation, Kyoto, Japan). p-Cresol was eluted isocratically using acetonitrile/water 40∶60 (v/v) as mobile phase (flow rate 1.0 ml/min) and measured using a Waters 470 fluorescence detector set at the excitation wavelength of 275 nm and at the emission wavelength of 300 nm. Under these conditions the retention time of p-cresol was 8.6±0.30 min. Peak identity was confirmed by mass spectrometry analysis using an API 3000 triple quadrupole mass spectrometer (Applied Biosystems, Canada) equipped with a Turbo-Ion Spray source. The HPLC system was calibrated by using standard solutions prepared by diluting p-cresol in methanol to the final concentrations of 13.5, 27.0, 54.0, 108.0, and 215.0 ng/ml. Under our experimental conditions, the limits of quantification (signal/noise ratio  = 10) and of detection (signal/noise ratio  = 3) of the technique, as evaluated on synthetic standards, were 0.060 and 0.017 ng/ml, respectively.

All chemicals and reagents were of either analytical or HPLC grade and were purchased from Delchimica (Naples, Italy). p-Cresol standard (minimum purity 99%) was from Sigma-Aldrich (Milan, Italy).

### Statistical analysis

Data were analyzed using SPSS 12.0 (SPSS Inc, Chicago, IL) and Stata12 (Stata corp, College Station, Texas ) setting the threshold for statistical significance at p values <0.05. The Shapiro-Wilk test was performed to assess whether the different sets of clinical and biochemical data were normally distributed or not. Normally distributed data are presented as means ± standard deviation (SD) whereas skewed data are reported as median with interquartile range (IQR). Statistical comparisons among the three experimental groups (no binder, lanthanum and *Sev*) were carried out using univariate ANOVA followed by the Bonferroni post-hoc test for normally distributed variables and Kruskal-Wallis test followed by Dunn's post-hoc test for skewed data. Categorical variables were expressed as percent and analyzed by χ^2^ test.

Multiple linear regression analysis was performed to identify factors that were independently correlated with plasma p-cresol concentrations. Specifically, a general linear model (GLM) was built using a stepwise method and setting p-cresol plasma concentration as the dependent variable and the following as independent variables: total Kt/V, total clearance, rGFR, urine volume, presence or absence of diabetes, serum albumin, serum phosphate and serum iPTH. The values of p-cresol concentrations were ln-transformed before inclusion into the model because they were not-normally distributed. Post-hoc statistical power analysis for linear regression models was performed according to the method of Cohen [23]. Pearson correlation was used to analyze the correlation between ln plasma p-cresol concentration and dose of *Sev*.

## Results

**Figure 1:**
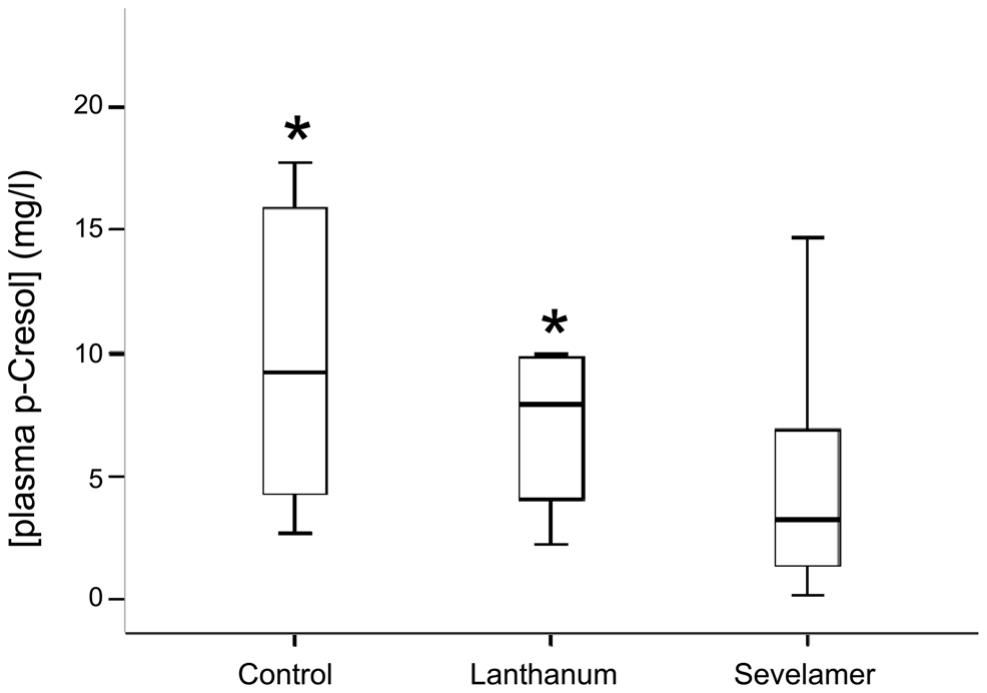
Box and whiskers plot of serum p-cresol levels in patients assuming *Sev*, lanthanum or no binder. The bars represent median, 25^th^ and 75^th^ percentile of plasma p-cresol. * p<0.05 vs no binder at Dunn's post-hoc test.

**Figure 2:**
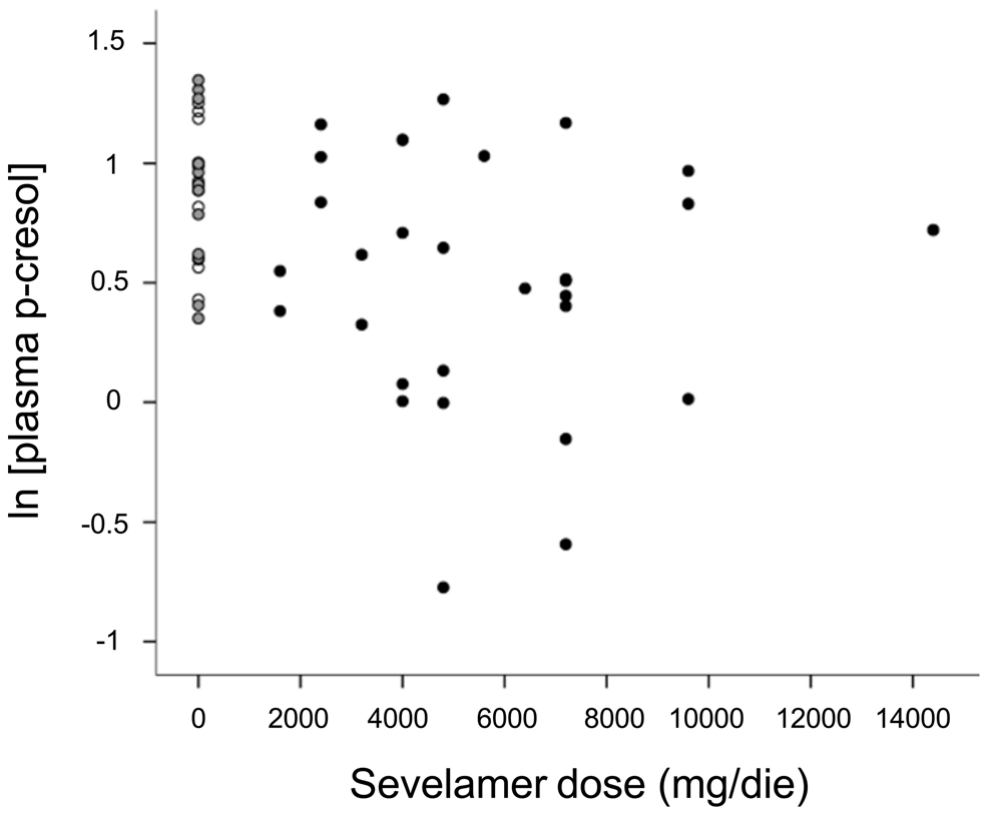
Scatter plot of serum p-cresol concentrations as a function of *Sev* daily dosage. The white circles correspond to patients of the no-binder group, the gray circles to those assuming lanthanum and the black circles to subjects assuming *Sev*.

57 ESRD patients receiving PD and attending the Division of Nephrology of the Federico II University of Naples as outpatients were recruited for the study. Fifteen of them were women (26%) and the remaining 42 men (74%). At the time of study, patients had a mean age of 59.7±14.5 years. Forty-one patients were on continuous ambulatory peritoneal dialysis and 16 on automated peritoneal dialysis. Based on whether they assumed or not phosphate binders and on which binder they assumed, the patients were stratified in three groups: no binder (n = 12), lanthanum (n = 16) and *Sev* (n = 29). 20 patients (*Sev*  = 10, Lanthanum  = 5, No binder  = 5) were treated with calcitriol (0.25 µg every other day–0.5 µg/day) and 23 (*Sev*  = 13, Lanthanum  = 5, No binder  = 5) with paricalcitol (1 µg every other day–1 µg/day). Because the therapy with hypophosphatemic drugs was individually tailored to achieve target plasma PO_4_ concentrations, *Sev* and lanthanum were administered at different dosages in different subjects (dosage ranges: 1600–14400 and 750–3000 mg/die for *Sev* and lanthanum, respectively). There was no significant difference among the different groups neither in mean age at the time of the study, nor in mean body weight, or relative percentage of the two sexes. Also peritoneal dialysis *vintage* (i.e. the length of time on dialysis in months) was similar in the three groups. Its mean in the whole patient population was 25.4±22.1 months and in all cases it was longer than six months. Six patients in each of the three patient groups were diabetic. No difference was observed among *Sev* , lanthanum and no binder groups in mean percentage of HbA1c . It was <7% in both groups suggesting that a good glycemic control was obtained both in all patient groups [24]–[25]. All data we reported so far suggest that *Sev*-treated, lanthanum-treated and no binder patients are very similar in their demographic, clinical and laboratory profile (Table 1 and Table 2). Nevertheless, significant differences emerged when we compared total p-cresol plasma concentrations. Plasma levels of this uremic toxin were significantly lower in *Sev* than lanthanum or no-binder groups [median and IQR: 3.3 (1.4–6.9) vs 7.9 (4.1–9.8) and 9.2 (4.3–15.9) in *Sev*, lanthanum and no binder groups, respectively; H = 9.6, p<0.008] (Fig. 1 and Table 2). In addition, in *Sev*-treated patients plasma p-cresol concentration was linearly related to the dose of the PO_4_ binder assumed by the patient being higher *Sev* doses associated to lower concentrations of this uremic toxin (r = −0.319; P = 0.025) (Fig. 2). Another relevant difference was observed in hs-CRP concentrations that were significantly lower in *Sev* than in lanthanum or no-binder groups (median and IQR: 3.8 (1.2–6.6) vs 6.3 (2.6–10.0) and 5.9 (3.4–8.4) in *Sev*, lanthanum and no binder groups, respectively; H = 10.2, p<0.006) (Table 2). No significant difference was observed neither in total creatinine clearance, weekly Kt/V, rGFR and urine volume suggesting that residual renal function and dialysis efficiency were similar in these three groups (Table 1). Moreover, also serum albumin concentrations were not significantly different among the three groups suggesting that the differences in the plasma concentration of p-cresol, a uremic toxin that circulates largely bound to serum albumin, could not be explained by a lower protein-bound fraction (Table 2). Considering that *Sev* therapy was started because of concurrent hyperphosphatemia and that the main pharmacological effect of *Sev* is to lower PO_4_, we compared PO_4_ circulating levels in the three groups (Table 2). No difference among groups was found (Table 2) suggesting that the treatment with the PO_4_ binder was effective in normalizing PO_4_ profile.

**Table 1:** Demographic data and dialysis and clinical parameters of sevelamer,lanthanum and no-binder patient groups.

	All	Sevelamer	Lanthanum	No binder	p
Patients (n.)	57	29	16	12	
Male Gender (%)	73.7	72.4	81,2	66,7	0.591
Age (years)	59.7±14.4	60.3±16.1	56.8±14.4	63.8±7.6	0.533
Diabetes (%)	33	28	31	50	0.375
Body Weight (kg)	76.3±14.1	76.8±12.7	77.2±15.5	73.1±17.5	0.778
Dialysis vintage (months)	20 (6–32)	12 (6–32)	27 (22–33)	12 (6–26)	0.082
Total clearance (L/week/1.73 m^2^)	64.8 (54.8–84.9)	71.3 (58.7–84.4)	61.3 (54.8–69.1)	70.0(49.8–90.4)	0.265
Renal clearance (L/week/1.73 m^2^)	30.0 (4.9–44.5)	32.7 (0.0–49.2)	22.6 (3.1–40.7)	32.5 (11.1–41.9)	0.580
Dialysis clearance (L/week/1.73 m^2^)	37.0 (30.0–48.4)	36.0 (29.4–52.0)	39.1 (30.0–47.9)	40.6 (32.5–46.7)	0.745
Total weekly Kt/V	1.86 (1.68–2.22)	1.9 (1.69–2.24)	1.73 (1.41–1.93)	2.11 (1.75–2.44)	0.119
Renal weekly Kt/V	0.67 (0.14–1.09)	0.67 (0.00–1.18)	0.57 (0.07–1.08)	0.87 (0.34–1.39)	0.682
Dialysis weekly Kt/V	1.14 (0.81–1.65)	1.12 (0.79–1.72)	1.12 (0.78–1.61)	1.37 (1.02–1.60)	0.808
Residual GFR (ml/min per 1.73 m^2^)	2.8 (0.4–4.0)	2.8 (0.0–5.1)	2.5 (0.3–3.8)	2.7 (0.5–5.4)	0.737
nPCR (g/kg/day)	0.97±0.2	0.95±0.2	1.01±0.2	0.98±0.2	0.684
Urine volume (ml/day)	1200 (650–1850)	1200 (600–1800)	1100 (625–1800)	1350 (619–2470)	0.589

The data shown in the table are expressed as mean ±SD in the case of normally distributed variables or as median (IQR) in the case of skewed data, respectively. Categorical data are reported as percent values. The last column on the right reports the p values of ANOVA or Kruskal-Wallis test in the case of normally distributed and skewed data, repectively.

**Table 2:** Biochemical parameters of sevelamer, lanthanum and no-binder patient groups.

	All	Sevelamer	Lanthanum	No binder	p
Plasma p-cresol (mg/l)	5.3 (2.8–10.1)	3.3 (1.4–6.9)	7.9 (4.1–9.8)*	9.2 (4.3–15.9)*	0.008
Serum hs–CRP (mg/l)	5.8 (2.6–6.6)	3.8 (1.2–6.6)	6.3 (2.6–10.0)*	5.9 (3.4–8.4)*	0.006
Serum phosphate (mg/dl)	4.7±1.0	4.5±1.0	5.1±1.0	4.4±0.7	0.107
Serum calcium (mg/dl)	9.1±0.7	9.2±0.6	9.1±0.9	8.9±0.6	0.471
iPTH (pg/ml)	196 (106–310)	152 (78–256)	273 (141–334)	251 (177–481)	0.039
Albumin (g/dL)	3.8±0.4	3.7±0.4	3.9±0.5	3.8±0.4	0.568
Total cholesterol (mg/dL)	164±54	151±41	180±79	180±27	0.170
HDL cholesterol (mg/dL)	45±16	45±13	49±21	39±11	0.332
Triglycerides (mg/dL)	126 (106–151)	118 (102–151)	125 (111–142)	174 (133–191)	0.177
Hemoglobin (g/dl)	11.4±1.0	11.2±1.1	11.4±0.9	11.8±1.0	0.328
HbA1c (%)	6.0±1.2	5.9±0.9	6.2±1.6	6.1±1.1	0.750

As in Table 1, the data shown are expressed as mean ±SD in the case of normally distributed variables or as median (IQR) in the case of skewed data, respectively. The last column on the right reports the p values of ANOVA or Kruskal-Wallis test in the case of normally distributed and skewed data, respectively. The asterisks indicate a statistically significant difference at the level of p<0.05 versus *Sev* group as calculated by Dunn's post hoc test.

To identify the factors associated to p-cresol plasma concentrations we performed a stepwise multiple regression analysis. As shown in Table 3, the only variable significantly associated to plasma p-cresol was whether the patient assumed or not *Sev* (R^2^ = 0.19; p = 0.001 with a statistical power of 0.90). On the contrary, we did not observe any significant association between plasma concentrations of this uremic toxin and either urine volume, rGFR, total Kt/V, total clearance, nPCR, presence or absence of diabetes, serum albumin, serum phosphate and serum iPTH concentrations.

**Table 3:** Stepwise multiple regression analysis of ln p-cresol plasma concentrations in peritoneal dialysis patients.

Parameter	β-coefficient	Standard Error	p
Sevelamer Use	−0.90	0,405	0.001
Intercept	2.95	0.255	<0.0001

*Sev* use was the only variable left in the model because significant at the 0.10 level. The following variables of the original model were excluded because they did not meet the 0.10 significance level in the stepwise procedure: total clearance (β = 0.04, p = 0.77), rGFR (β = −0.07; p = 0.57), urine volume (β = 0.02; p = 0.88), total Kt/V (β = −0.06; p = 0.65), presence or absence of diabetes (β = −0.07; p = 0.55), nPCR (β = 0.01; p = 0.95), serum albumin (β = 0.003; p = 0.98), serum phosphate (β = −0.03; p = −0.82) and serum iPTH (β = −0.11; p = 0.40).

## Discussion

The main finding of the present study is that in PD patients the concomitant use of Sev for hyperphosphatemia is associated with lower plasma p-cresol concentrations. This was suggested by the significantly lower plasma p-cresol concentrations in patients assuming Sev as compared with those assuming no binder or lanthanum and was confirmed by multiple linear regression analysis.

How could the association between Sev use and lower plasma p-cresol concentrations be explained? Because of its experimental design our study does not allow to establish any causal relationship between the variables that were examined. However, some working hypotheses can be considered. Reasonable hypotheses are that in our series patients assuming Sev could have lower protein intake, a better renal function or lower serum albumin concentrations. Indeed, protein intake, is a limiting factor for the production by gut resident bacteria of p-cresol [26], that circulates in plasma bound to serum albumin and is mainly removed from the blood by the kidney [27]. However, we can reasonably exclude all these hypotheses because we did not observe any statistically significant difference among the different groups in the aforementioned variables. Moreover, neither nPCR or serum albumin or related parameters of renal function such as urine volume or rGFR predicted plasma p-cresol concentrations in our GLM. Similarly, although previous studies showed that diabetes could per se cause an increase in plasma p-cresol level [28]–[29], the prevalence of diabetic patients was not significantly different in our groups of patients and diabetes did not predict p-cresol concentrations in our GLM. In our opinion, the more reasonable hypothesis to explain our findings is, instead, that Sev could somehow directly lower p-cresol plasma concentrations. This possibility is supported by the observation that in the Sev group, plasma p-cresol concentrations were inversely related to the daily dose of the drug assumed by each patient, suggesting that the effect could be dose-related. However, the mechanism by which Sev could lower p-cresol concentration remains unclear. The main pharmacological effect of Sev is its ability to bind PO_4_ ions in the gut, therefore preventing their absorption and, ultimately, lowering their concentrations in the plasma [18]. However, the following considerations suggest that it is unlikely that the Sev affects p-cresol plasma concentrations by lowering serum PO_4_. First, a group of our patients assumed another PO_4_ lowering drug, lanthanum, but its p-cresol plasma concentrations were similar to those found in the group of subjects not assuming PO_4_ binders and significantly higher than in the group of subjects receiving Sev. Second, mean serum phosphate and calcium concentrations were similar in the different groups of patients and in all cases they were in the ranges recommended by the KDIGO guidelines [30]. Similarly, iPTH levels were in the normal range both in patients treated with Sev or lanthanum and in those not assuming PO_4_ binders although the values measured in the latter two groups were slightly but not significantly higher. All these considerations seem to exclude that *Sev* could affect p-cresol levels by its main action on PO_4_ intestinal absorption.

A mechanism that could instead explain our findings is the binding of p-cresol to Sev in the gut. This appears an intriguing possibility considering that it has been shown that Sev has the ability to sequester several uremic toxins in the intestinal lumen and, therefore, to prevent their absorption and lower their circulating concentrations [31]–[33]. Importantly, this effect has not been observed with other PO_4_ binders like lanthanum and, therefore, it does not seem to be a general characteristic of PO_4_ binders but a specific property of Sev. However, while there is a strong evidence of a direct binding of indoxyl-sulphate and urate to Sev [31]–[32], the hypothesis that also p-cresol could bind to this drug has been controversial so far. The binding of this toxin to Sev has been, indeed, demonstrated in vitro [21], whereas no effect was observed in a mouse model of CKD [33]. In the only study performed in human patients [21], Sev was found ineffective in lowering the plasma concentration of p-cresol in a series of 57 hemodialysis patients. The reason of the differences between these results and ours are unclear but they could be related to the different dialysis procedure that was used. Recent evidence suggests, indeed, that the underperfusion that can take place during extracorporeal hemodialysis could alter the permeability of the intestinal barrier favoring the passage of intestinal bacterial products (Intestinal-Renal Syndrome) [34]. Supporting this hypothesis, McIntyre et al. recently reported [35] that measurable levels of endotoxins coming from intestinal bacteria can be detected in the plasma of hemodialysis patients and, importantly, that they increase at the time of dialysis. These important results have been interpreted assuming that dialysis-induced hemodynamic stress could damage the intestinal mucosa finally leading to an increase of its permeability to endotoxins [35]. Although a formal demonstration of this hypothesis by quantitative measurement of intestinal blood flow is still lacking [34], it is tempting to speculate that Sev could be less effective in preventing p-cresol absorption in hemodialysis patients because this dialysis procedure increases the permeability of the intestinal mucosa to the point that too much p-cresol can escape from Sev and enter the blood.

The present cross-sectional observational study suggests that Sev could be an effective therapeutic strategy to lower p-cresol concentrations in PD patients. If confirmed in larger randomized double blinded clinical trials, this observation could have important implications for planning the best pharmacological treatment in PD patients. The concept that lowering p-cresol concentrations is a priority in CKD patients strongly emerged, indeed, during the last years because of compelling experimental evidence showing that this uremic toxin negatively influences the prognosis of this disease by significantly increasing the cardiovascular risk [34], [36]. Unfortunately, no pharmacological treatment effective in lowering p-cresol is available with the only exception of AST-120, a non-absorbable carbon adsorbent approved in Japan that adsorbs both indoxyl-sulphate and p-cresol in the gut [37]–[38]. Sev could be a good therapeutic choice to lower plasma p-cresol also considering that, besides lowering p-cresol, it could also favorably impact on other cardiovascular risk factors in PD patients. To be specific, Sev is known to decrease serum C-reactive protein [31], [39]–[45], a well known predictor of cardiovascular risk [46], and this was also observed in our PD patient series. This effect has been attributed to a lower absorption of intestinal endotoxins that causes a decrease in systemic endothelial damage and its related inflammatory response [47]–[49]. Moreover, it has been reported that Sev decreases HbA1C and LDL and increases HDL and plasma concentrations [39]–[41], [43], [50]–[51]. Consistent with the ability of Sev to favorably affect cardiovascular risk factors, a significant decrease in all case and cardiovascular mortality has been recently reported in a cohort of hemodialysis patients as compared with patients with a similar impairment in renal function assuming calcium carbonate or no PO4 binder [52].

The main limitation of this study is in its observational design. Even though we carefully looked at the homogeneity of the groups and, indeed, no significant difference was observed between them in main demographic, clinical or laboratory characteristics, further double-blinded randomized studies will be mandatory to confirm our findings.

In conclusion, we reported evidence that *Sev* could lower p-cresol levels in PD patients. Considering that *Sev* also decreased hs-CRP level, our results suggest that this drug could have an indication in CKD patients independent from its hypophosphatemic effect and related to its ability to reduce the high cardiovascular risk of these subjects by multiple mechanisms also including a decrease in p-cresol plasma levels.
